# Cardiac involvement in anti-MDA5 dermatomyositis: a case-based review

**DOI:** 10.1007/s10067-022-06401-x

**Published:** 2022-12-01

**Authors:** Diana C. Quintero-González, Kevin Navarro-Beleño, L. V. López-Gutiérrez, Marcela Muñoz-Urbano, Adriana-Lucía Vanegas-García, Carlos Horacio Muñoz-Vahos

**Affiliations:** 1 Rheumatology Section, Universidad de Antioquia, Hospital San Vicente Fundación, Medellín, Colombia; 2 Internal Medicine Department, Universidad de Antioquia, Hospital San Vicente Fundación, Medellín, Colombia

**Keywords:** Anti-melanoma differentiation-associated gene 5 antibody, Cardiac disease, Dermatomyositis

## Abstract

Among myositis-specific antibodies, anti-melanoma differentiation-associated gene 5 (anti-MDA5) is one of the antibodies with a unique spectrum that is expressed principally in clinically amyopathic dermatomyositis (CADM) and, to a lesser extent, in dermatomyositis (DM). In addition to muscle and classical skin involvement, patients with anti-MDA5 DM/CADM are characterized by the expression of rapidly progressive interstitial lung diseases, vasculopathic lesions, and non-erosive arthritis. Although cardiac involvement has been described in other inflammatory myopathies, such as myocarditis, pericarditis, and conduction disorders, in anti-MDA5 DM/CADM patients, heart disease is infrequent. We report a case of a young male presenting with constitutional symptoms, polyarthritis, skin ulcers, and mild muscle weakness who developed an episode of high ventricular rate atrial fibrillation during his hospitalization. The anti-MDA5 DM diagnosis was supported by increased muscular enzymes, positive anti-MDA5 and anti-Ro52 antibodies, and the presence of organizing pneumonia. He was treated with high-dose glucocorticoids, rituximab, and beta-blocker drugs and received pharmacological cardioversion, which improved his myopathy symptoms and stabilized his heart rhythm. Here, we describe eight similar cases of anti-MDA5 DM/CADM with cardiac involvement. The case presented and the literature reviewed reveal that although rare, physicians must be aware of cardiac disease in patients with suggestive symptoms to guarantee early assessment and treatment, thereby reducing life-treating consequences.

## Introduction

Idiopathic inflammatory myopathies (IIMs) are a group of autoimmune diseases with a broad clinical spectrum characterized mainly by muscle and other organ involvement, such as the skin, joints, heart, and lungs [1]. The anti-melanoma differentiation-associated gene antibody (anti-MDA5) is a myositis-specific autoantibody (MSA) against a 140-kDa polypeptide described by Sato et al. in 8 Japanese patients with clinically amyopathic dermatomyositis (CADM) [2]. Further description found that patients with anti-MDA5 have a unique clinical phenotype that includes CADM or classical dermatomyositis (DM), rapid progressive interstitial lung disease (RP-ILD), arthritis, and vasculopathy lesions [3, 4]. Anti-MDA5 DM/CADM represents approximately 40% of DM cases in children [5] and 10 to 25% of adult patients with DM [6, 7]. The mortality rate in this disease depends on its clinical phenotype; therefore, patients with RP-ILD have a worse prognosis [8]. Cardiac involvement is rare and has been scarcely reported in the literature. We described a case of a young male with anti-MDA5 DM with new-onset atrial fibrillation (AF).

## Methods

We reviewed the literature regarding heart involvement in anti-MDA5 DM/CADM in MEDLINE, EMBASE, SCOPUS, and LILACS until August 2022. We selected English- and Spanish-language-related case reports. Key terms used in the research were “heart disease,” “cardiac disease,” “MDA5,” and “dermatomyositis.” We selected eight articles [8–16] that reported cardiac manifestations in patients with anti-MDA5 DM/CADM (Table 1).

**Table 1:** Clinical and demographic characteristics of patients with cardiac involvement in MDA5 dermatomyositis

Author, year, country	Age (year), gender	Joints	Myopathy	Skin lesions	Pneumopathy	Cardiovascular symptoms	Cardiac disease	Anti-SS-A/Ro52 Ab	Studies	Echocardiogram	Cardiac MRI/endomyocardial biopsy	Treatment	Outcome
Gupta R, 2020, Tonga [15]	F, 27	+	+	Heliotrope erythema	ND	Palpitations	Myocarditis, sinus tachycardia	ND	Negative troponin	EF 42%	Left ventricular dilatation, EF 42%. T2 black blood, T1 mapping, and LGE images	Immunoglobulin, cyclosporine, high-dose steroids	Improve
Pau-Charles I 2013, Philippines [9]	M, 55	+	-	Livedo reticularis, skin necrosis	ND	Cardiogenic shock	Myocarditis, pericarditis, third-degree atrioventricular block	ND	Normal coronary angiography	EF 25%, global motility dysfunction, moderate mitral regurgitation	ND	Azathioprine, prednisone, cyclophosphamide, immunoglobulin Vasopressor, inotropic, nifedipine, digoxin	Deceased
Allenbach Y, 2020, France (2 cases) [7]	ND	ND	ND	ND	ND	Severe heart failure	Myocarditis	ND	ND	ND	ND	ND	ND
Ma X, 2021, China [13]	F, 43	ND	+	ND	ND	Heart Failure	Left anterior fascicular block, frequent ventricular ectopy	+	Positive troponin, NT proBNP 4526 pg/ml	Left ventricular dilation. EF 25%. Increased filling pressures E / e mean ratio 32. No valve disease	Atrophic and disordered myofibers, interstitial fibrosis without inflammatory infiltrates IHC overexpression of polyubiquitin binding protein p62 / SQSTM1 protein and positivity of cleaved-caspase 3 in cardiomyocytes	Prednisone, tacrolimus, methotrexate Heart transplant	Myopathy improved; cardiomyopathy aggravated until transplantation
Kurtzman DJB, 2018, USA [11]	F, 30	+	+	Ulcers on palms and soles, keratotic plaques on arms and elbows	Airspace disease, pleural effusion, and restrictive disease	Dyspnea	Myocarditis, pericarditis	ND	ND	ND	Pericardial effusion, inflammation, and myocardial edema	Steroids, azathioprine	Improve
Ryan ME, 2021, USA [10]	M, 14	+	-	-	ND	ND	Intermittent second-degree AV block	ND	proBNP 820 pg/ml, Normal troponin	ND	ND	Methotrexate, prednisone, methylprednisolone, monthly immunoglobulin	Improve, and first-degree AV block resolved
Sakamoto N, 2019, Japan [14]	M, 68	ND	ND	No	Interstitial pneumonia	ND	Complete AV block	ND	ND	ND	ND	Methylprednisolone, tacrolimus, cyclophosphamide	Deceased
Sakurai N, 2011, Asia [8]	M, 9	ND	+	Gottron´s papules	Interstitial pneumonia, pneumomediastinum, pneumothorax	ND	Sinus tachycardia 150 bpm, T wave flattening	ND	ND	EF 44%	ND	Cyclophosphamide	Deceased
Quintero-González DC, 2022, Colombia	M, 17	+	+	Biphasic Raynaud phenomenon and skin ulcers	Organizing pneumonia	Chest pain	Atrial fibrillation 152 bpm	+	Normal I troponin	EF 54%	LGE	3 pulses of methylprednisolone followed by prednisolone, methotrexate, and rituximab Metoprolol, amiodarone	Improve, and the ventricular response was controlled

*BMB*, bone marrow biopsy; *bpm*, beats per minute; *IHC*, immunohistochemistry; *HRCT*, high-resolution computed tomography; *CT*, computed tomography; *yr*., years; *ND*, non-data; *LGE* long gadolinium enhancement

### Case presentation

**Fig. 1:**
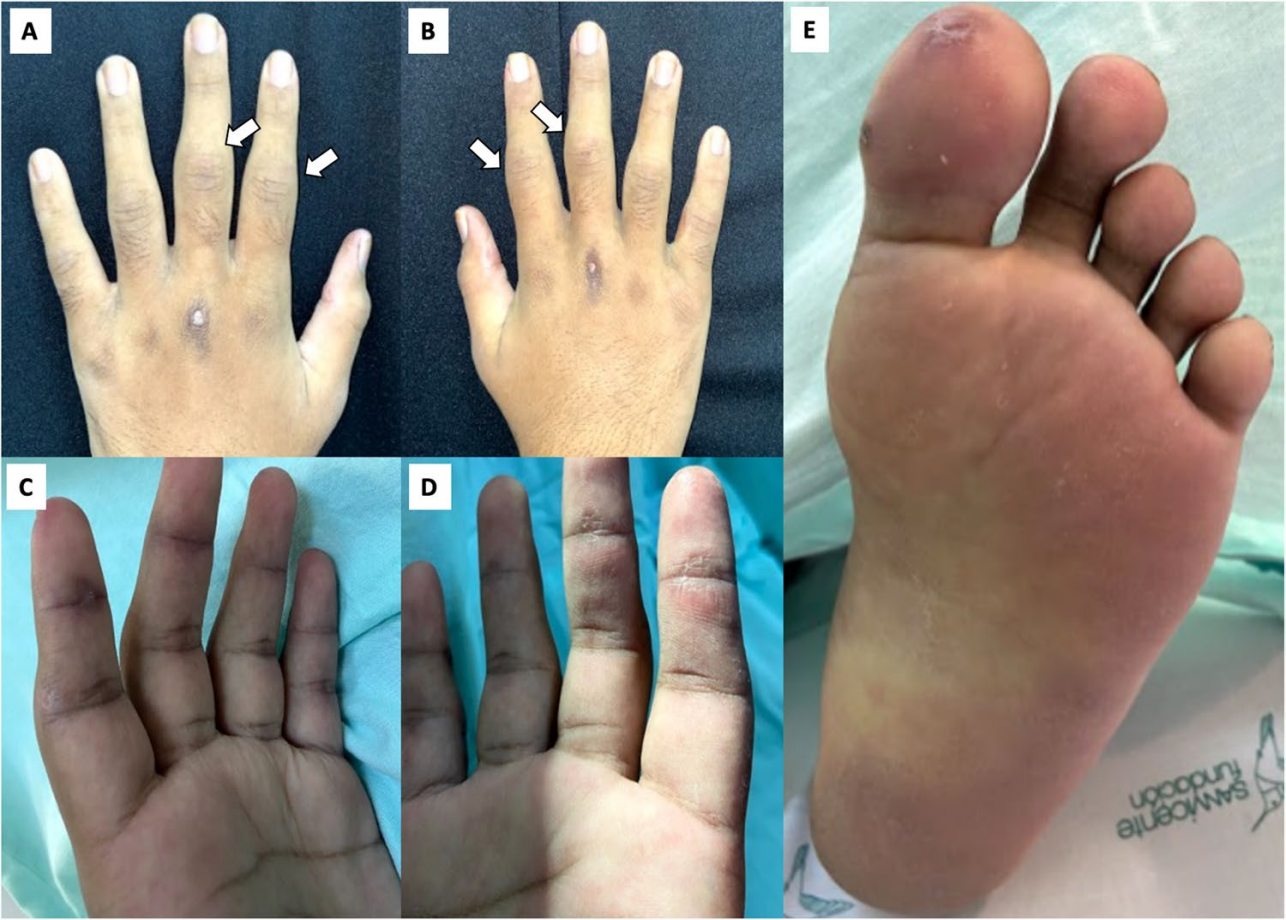
**A**, **B** Brownish-erythematous plaques with a residual scar of ulcers on the bilateral third metacarpophalangeal joints and arthritis on the bilateral second and third proximal interphalangeal joints (white arrows). **C**, **D** Palmar papules on the distal interphalangeal joints. **E** Violaceous macule on the tip of the hallux pulp

A 17-year-old male, previously asthmatic, presented to the rheumatologist with an 8-month history of polyarthritis, prolonged morning stiffness, fever, and 9-kg weight loss. Because the main clinical suspicion was rheumatoid arthritis, he was initially treated with methotrexate (15 mg QW) and prednisolone 10 mg QD without achieving a complete response. During follow-up, he described new-onset proximal muscle weakness, biphasic Raynaud’s phenomenon, and skin ulcers for 3 months, for which he was referred to the emergency department. Physical examination showed arthritis in the elbows, wrists, and several metacarpophalangeal and proximal interphalangeal joints with slight proximal upper limb weakness. Skin examination revealed diverse lesions: (1) hyperpigmented plaques on elbows, (2) brownish-erythematous plaques with a residual ulcer scar on joints in the hands, (3) palmar papules on the distal interphalangeal joint, and (4) violaceous macules on the tip of the hallux pulp (Fig. 1).

**Fig. 2:**
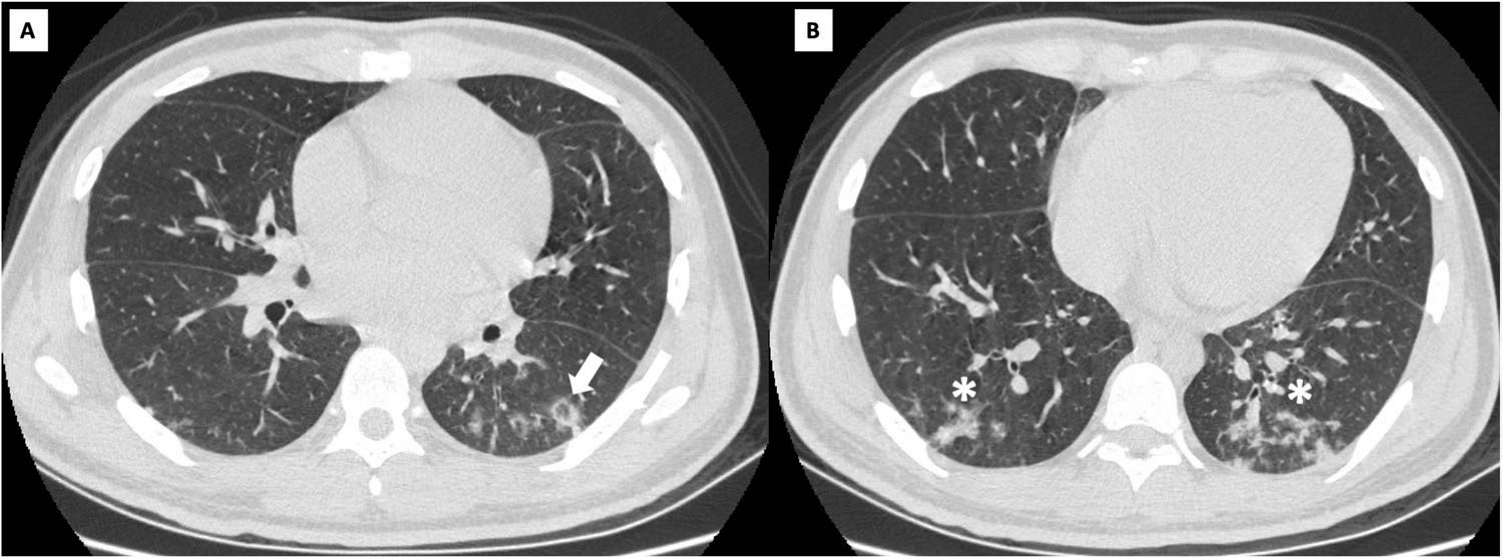
High-resolution chest tomography shows **A** scant reticulation and reversed halo sign in the left lower lobe (white arrow) and **B** small peribronchial and subpleural consolidation (white asterisk)

Regarding laboratory findings, the most relevant was the increase in acute phase reactants, mild normocytic anemia, and a minimal muscle enzyme elevation with a creatinine kinase level of 237 U/L (upper limit of normal [ULN] 171 U/L) lactic dehydrogenase 500 mg/dL (ULN 460 mg/dL), aspartate aminotransferase 59 mg/dL (ULN 45 mg/dL), and alanine aminotransferase 40 mg/dL (ULN 40 mg/dL). Skin biopsy revealed thrombotic vasculopathy and interphase dermatitis changes (Fig. 2). After ruling out other differential diagnoses, such as malignancy and infection, an MSA and myositis-associated autoantibody panel was performed. Antinuclear antibodies were positive with a cytoplasmic pattern, and anti-Ro52 and anti-MDA5 antibodies were present in low and high titers, respectively. Finally, the complete clinical picture allowed the diagnosis of anti-MDA5 DM.

**Fig. 3:**
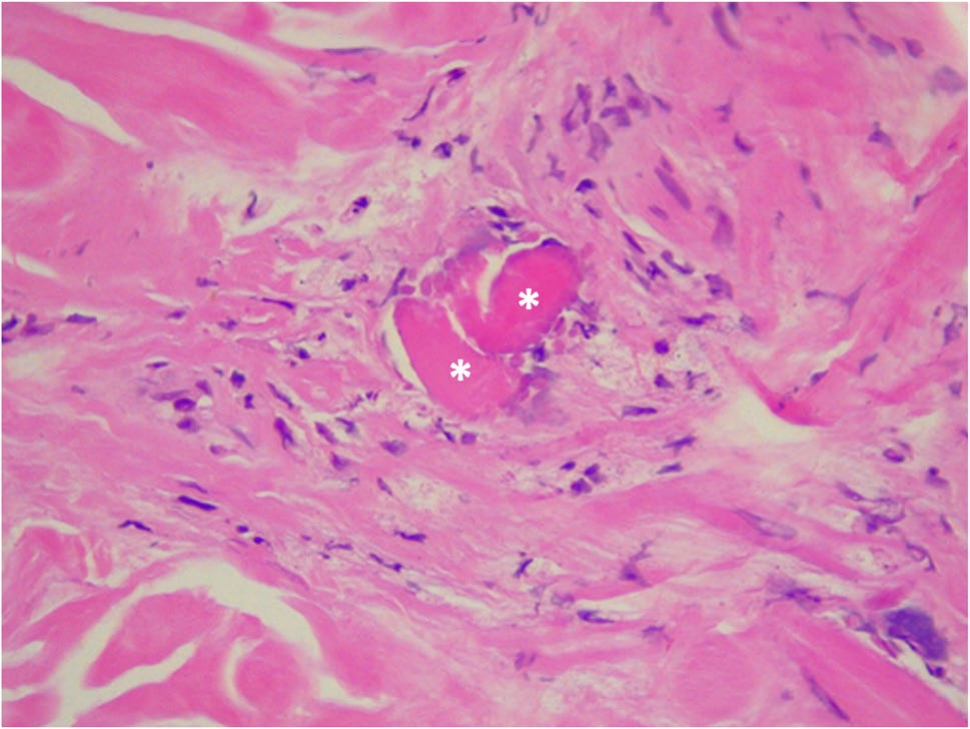
On the dermis, there is evidence of a small vessel with a fibrin thrombus in its lumen (white asterisks), extravasated erythrocytes, and surrounding chronic inflammatory infiltrate, hematoxylin and eosin 40 ×

Although the patient had no clinical findings suggestive of respiratory distress, high-resolution computed tomography, performed as part of the screening imaging, revealed subclinical interstitial lung involvement in organizing pneumonia (OP) pattern (Fig. 3). Due to the predominance of articular symptoms, the patient was classified as Cluster 3 of the clinical phenotypes described by Allenbach [8]. Initial treatment consisted of methylprednisolone pulses (500 mg QD for three days) followed by prednisolone 1 mg/k/d and methotrexate 15 mg QW.

One day after glucocorticoid pulse therapy, the patient presented oppressive chest pain at rest, with tachycardia (152 beats per minute) and normal arterial blood pressure. An electrocardiogram (ECG) was performed during the episode showing an atrial fibrillation (AF) pattern. Therefore, he received intravenous metoprolol and pharmacologic cardioversion with amiodarone, which stabilized the heart rate and resolved the thoracic pain. Concerning cardiac assessment, troponin I and echocardiogram were normal, with a preserved left ventricular ejection fraction of 54%. Additionally, cardiac magnetic resonance (CMR) did not show late gadolinium enhancement (LGE) or structural alteration. Finally, 1 g of rituximab was prescribed on days 0 and 15 due to persistent and severe arthritis. At the 6-month follow-up, the patient reported a complete response of articular, muscular, and skin manifestations and a lack of cardiorespiratory symptoms or new high ventricular rate atrial fibrillation episodes.

## Discussion

Anti-MDA5 DM/CADM diagnosis presents challenges since vascular and joint manifestations can occur in other IIMs, such as antisynthetase syndrome and overlapping myositis. Physical examination is crucial for establishing differences between them; a clue in the assessment is the typical expression of vascular disease in the skin. In addition to Raynaud’s phenomenon (67%) and mechanic’s hands (29%) [12], patients have a unique cutaneous phenotype, with skin lesions such as ulcers (80%) and palmar papules (60%) [17]. Additionally, arthralgia and arthritis are characteristics of the disease and are present in 70% of patients [8]. ILD is seen in almost all cases, with the rapid progressive phenotype as the most regular expression (70%) [18] and OP as the characteristic tomographic pattern [19].

Considering that approximately two thirds of patients have CADM [8] and an increased risk of RP-ILD (OR 20.4, 95% CI 9.02–46.20) [20], thoracic imaging screening is mandatory to rule out pulmonary involvement. Regarding autoantibodies, severe cutaneous and lung involvement are associated with anti-Ro antibody positivity [21], and the increase in anti-MDA5 antibody levels has been considered as a predictor of RP-ILD flares [22]. The broad clinical spectrum allowed the identification by Allenbach et al. of three clusters (pulmonary, vasculopathic, and articular), with implications for prognosis [8]. Treatment strategies depend on clinical phenotype; those with RP-ILD benefit most from immunosuppressor combinations, such as glucocorticoids and calcineurin inhibitors, with or without cyclophosphamide [23]. For refractory disease, rituximab is an option that improves pulmonary symptoms, function tests, and imaging findings [24].

### The heart in immune inflammatory myopathies

Cardiac involvement in IIMs was first described in 1899 and was considered a rare presentation until the 1970s. However, more recent cohorts have shown an increase in its incidence [25, 26]. Heart disease has been reported in 9 to 75% of patients, with a wide prevalence margin due to patient selection, diagnostic tools, and the inclusion of subclinical involvement [26–28]. It is well recognized as a life-threatening manifestation [26, 29] and is considered by some authors to be the leading cause of death in IIMs [29, 30]. Although it is difficult to establish an accurate rate, mortality for this cause was reported in 10 to 20% of polymyositis (PM) patients [31].

The Euromyositis registry of 3067 cases of IIMs reported cardiac involvement in up to 9%, defined as pericarditis, myocarditis, arrhythmia, or sinus tachycardia due to the IIM disease process [32]. According to published literature, heart failure is the most common presentation observed in up to 45% of cases. Furthermore, a cohort study with a total of 1145 patients found an increased risk of coronary heart disease with hazard ratios of 2.21 (95% CI 1.64–2.99) in DM and 3.73 (95% CI 2.83, 4.90) in PM [33]. Unlike the described conditions, pericarditis and myocarditis are not frequent findings in IIMs; a prospective study of 26 patients found only three patients with pericarditis (12%), all with minimal and hemodynamically insignificant effusion [34]. A recent case–control study compared 31 cases of myocarditis with 31 IIM patients without cardiac involvement, finding that heart failure, arrhythmias, elevated troponin, and NT-proBNP could be relevant in the diagnosis [35]. Myocarditis in antisynthetase syndrome is described in 3.4% of cases and has no links to autoantibody specificity [36]. In the last two studies, there was a correlation between myocarditis and the presence of anti-mitochondrial M2 (AMA-M2) antibodies [35, 36].

Finally, arrhythmias such as AF also occurred, with a prevalence described in a Korean population with IIMs of 3.5%, which is higher than that in other autoimmune diseases (HR 3.29, 95% CI 1.94–5.58) [37]. Subclinical involvement is characterized by ECG abnormalities such as nonspecific changes in the ST-segment, left ventricular enlargement, and rhythm/conduction disorders [27, 38]. Two studies demonstrated an increased prevalence of ECG alterations; one reported these changes in 18% of IIM patients compared to 10% in healthy controls. In the second study, 50% of PM/DM patients had alterations versus 24% of controls (*p* = 0.008) [38].

The diagnostic approach to heart involvement requires biochemical tests, ECG, and imaging studies. From a systematic review, it can be concluded that a correlation exists between troponin levels in asymptomatic patients and the activity grade of IIMs and that the specificity of troponin I is better than that of troponin T levels [39, 40]. Concerning imaging tools, ventricular systolic function by echocardiography is usually normal; nonetheless, diastolic dysfunction is recurrently detected in IIMs [40, 41]. To date, novel CMR techniques are considered the most accurate myocarditis markers [42], where findings such as LGE areas (indicating scars or focal necrosis), abnormal T1, T2, and extracellular volume mapping are key and could be present despite a preserved ejection fraction [40]. Elevated cardiac biomarkers and reduced left ventricular ejection fraction are almost always present in overt myocarditis associated with IIMs [35, 40].

Chen et al. proposed an algorithm to assess heart disease in IIMs [43]. The first step is to perform troponin I level measurements, an ECG, and an echocardiogram; cardiac involvement is unlikely if these studies are normal [43]. However, if the troponin concentration is above the ULN and is not explained by other etiologies, CRM should be considered, and even myocardial biopsy in some cases [43]. Regarding immunosuppression, corticosteroid treatment outcomes in heart failure are controversial, and evidence is still insufficient [44, 45]. Severe cardiac complications should be treated according to current treatment standards.

### The heart in anti-MDA5 DM/CADM

Cardiac involvement in anti-MDA5 DM/CADM is infrequent, with only eight reported cases in the literature. As in IIMs, the clinical presentation is broad, ranging from myocarditis and pericarditis to cardiac conduction disorders (Table 1). Although heart disease is usually subclinical, patients can rapidly evolve, leading to life-threatening consequences [11]. The cases have a worldwide distribution without sex predominance and are slightly more frequent in Asia. The age of presentation varies between 9 and 68 years old, with a report of three patients of pediatric age. Most of the cases had symptoms of heart failure and other palpitations, and three of them did not have a description of the symptoms. Four of eight patients presented bradyarrhythmias, including symptomatic complete atrioventricular blocks [10, 11, 15] and tachyarrhythmias such as sinus tachycardia and atrial tachycardia [9, 16].

Fifty percent of cases had a diagnosis of myocarditis confirmed by CMR or histopathology [14, 16]. The ventricular tissues of one of the patients showed atrophic myofibers and disarranged myofibrils [14]. In immunohistochemistry, Ma et al. described significant interstitial fibrosis and positive staining of cleaved-caspase 3 in atrophic cardiomyocytes [14]. Surprisingly, high-sensible troponin was negative in most patients, even those with myocarditis. On the other hand, NT-proBNP was the most frequently increased, and cardiac biomarkers such as CK-MB were less frequently assessed [14]. Only one reported case presented with pericarditis and myocarditis, with worse outcomes [10]. Although MSAs are almost always mutually exclusive, one patient was positive for anti-MDA5 and antibodies against the signal recognition particle; in this case, the heart disease was better explained by the second [14]. Concerning other antibodies, none of the eight patients had anti-AMA M2 antibodies.

Regarding other diagnostic tools, Matsuo et al. recently published a retrospective analysis comparing ECG and echocardiography findings in IIM patients with or without anti-MDA5 antibodies (21 versus 41 cases). In this cohort, although only three patients had clinical heart disease, subclinical cardiac abnormalities were frequently reported in more than half of the patients. The results showed that anti-MDA5 DM/CADM cases had significantly lower T wave amplitudes on DI, DII, aVF, and V3 to V6 leads, which improved after treatment. In addition, on echocardiogram, they presented low E and A waves and decreased movement speed of the mitral valve annulus at the septum as a reflection of the underlying diastolic dysfunction [46]. Regarding the pathogenesis of cardiac involvement, two hypotheses were put forth; the first highlighted the harmful heart effects of some cytokines, such as IL-8 and IL-10, affecting calcium and potassium channels and inducing myocarditis [46]. The second hypothesis is based on the function of MDA5 as a pattern-recognition receptor for viral cytosolic RNA. To the best of our knowledge, coxsackievirus can trigger an anti-MDA5 DM/CADM by forming complexes that are recognized by antigen-presenting cells, inducing T and B cell immune responses and antibody production [4]. The fact that these viruses are also a cause of myocardial inflammation suggests that cardiac and muscle involvement share pathogenic pathways.

Another possible explanation is linked to the harmful potential of autoantibodies. It is known that patients with anti-MDA5 DM/CADM are frequently positive for anti-Ro antibodies (33%), especially for its polypeptide of 52 kDa (anti-Ro52). The presence of these antibodies, beyond being a random association because of their high frequency in other autoimmune diseases, in anti-MDA5 DM/CADM patients has been related to RP-ILD and vasculopathic lesions. In research by Temmoku et al., double-seropositive subjects had significantly lower survival based on Kaplan curves (*p* = 0.001), highlighting the worse outcomes [47]. Although the pathogenic pathways of anti-Ro52 antibodies in IIMs have not been described in the literature, a theory could be extrapolated from congenital cardiac block [48], where cardiac electrophysiological abnormalities are secondary to calcium dysregulation mediated by the molecular mimicry between anti-Ro antibodies and L-type calcium channels [49].

Finally, concerning outcomes, one patient had cardiogenic shock requiring intensive care unit admission for vasoactive support, and another underwent a heart transplant with important clinical improvement on follow-up [14]. The patients received aggressive immunosuppressive treatment with different schemes, with improvements in symptoms in more than 60% of the cases. Death was reported in 3 patients [9, 10, 15]. There is no specific recommendation about cardiac disease treatment due to the low frequency; therefore, immunosuppression is guided by other manifestations, mainly pulmonary involvement.

To our knowledge, this is the first case of atrial fibrillation associated with anti-MDA5 DM/CADM in a young male with a structurally healthy heart and without any other risk factors for developing arrhythmia. In conclusion, we highlight the relevance of considering cardiac disease in patients with anti-MDA5 DM/CADM and consistent symptoms. Although the clinical spectrum is heterogeneous, the most common presentation is myocarditis and conduction disorders (mainly tachyarrhythmias). To date, there is no strong evidence of the immunosuppression influence of heart involvement in this type of myopathy. Early suspicion, an accurate assessment, and specific treatment decrease the risk of life-threatening consequences and disease progression [12].
